# Supplementary material for: InPACT: a computational method for accurate characterization of intronic polyadenylation from RNA sequencing data
Source: Nat Commun. 2024 Mar 22;15:2583. doi: 10.1038/s41467-024-46875-8 (PMC10960005; doi:10.1038/s41467-024-46875-8)
Supplement: Supplementary file 1 — Supplementary Information [file 41467_2024_46875_MOESM1_ESM.pdf]

Supplementary Information

InPACT: A computational method for accurate characterization of intronic polyadenylation from RNA sequencing data

Xiaochuan Liu *et al.*

This PDF file includes:

Supplementary Fig. 1 to 14.

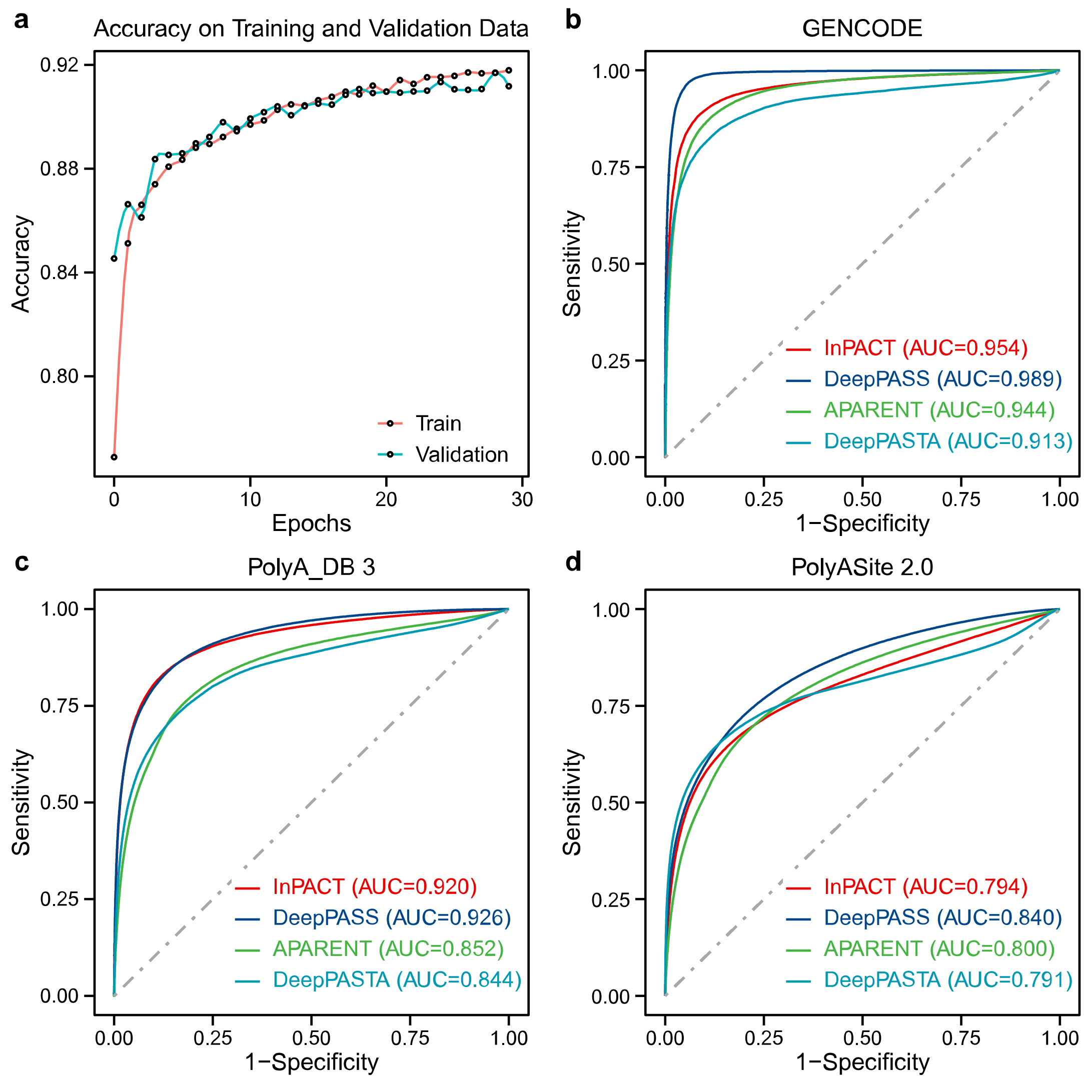

Supplementary Figure 1. Performance of the sequence module.

(a) Training and validation accuracy over 30 epochs of the CNN model.

(b-d) The receiver operating characteristic (ROC) curves show the performance of InPACT, DeepPASS, APARENT and DeepPASTA on three widely used polyA databases, namely GENCODE **(b)**, PolyA_DB 3 **(c)** and PolyASite 2.0 **(d)**. The area under ROC cure (AUC) values are indicated in each plot.

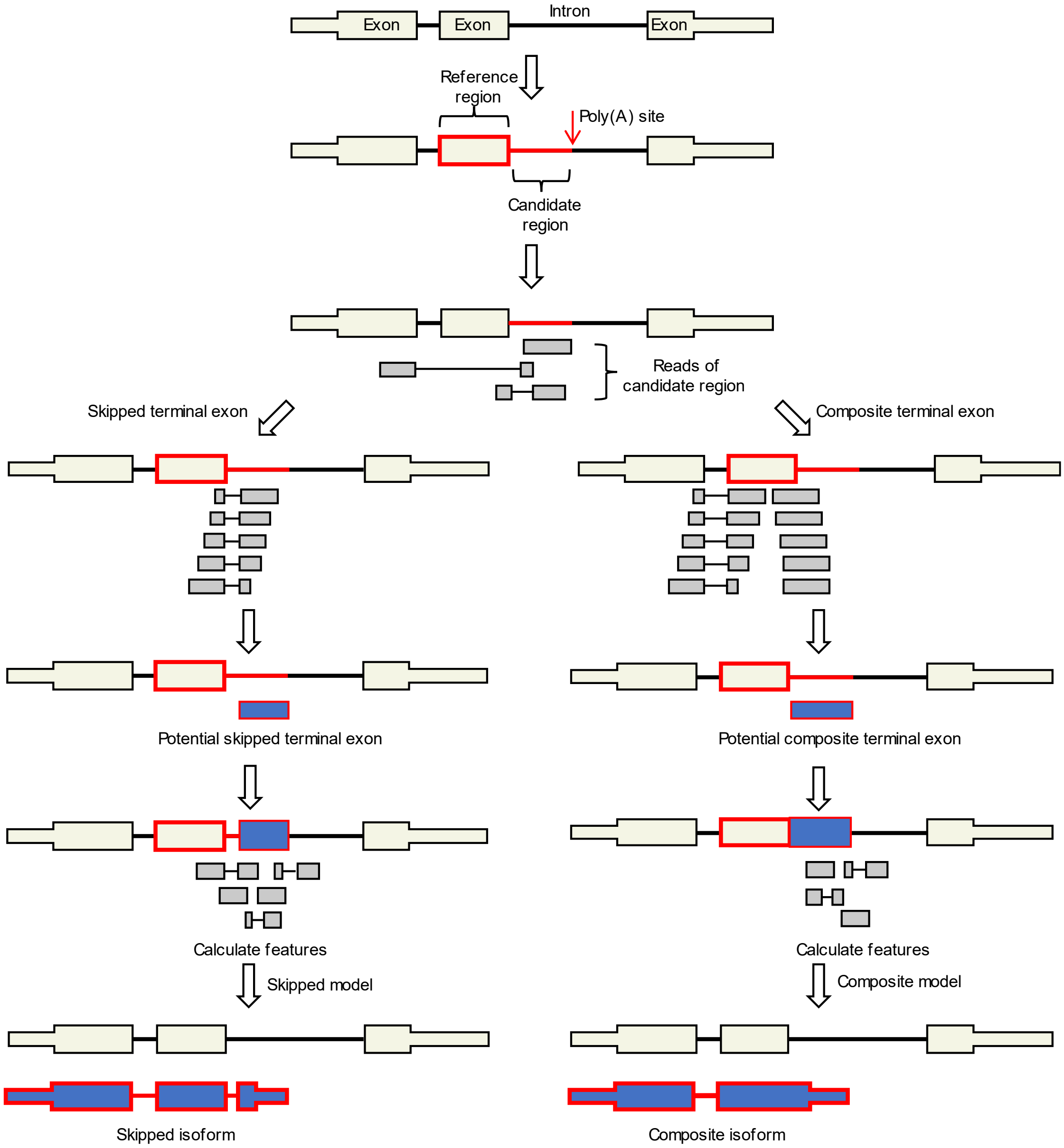

Supplementary Figure 2. Flow chart of constructing putative terminal exons from RNA-seq data.

To begin, select a putative polyA site located in introns with respect to the reference annotation. Candidate regions are then constructed from the polyA site to the closest annotated exons upstream, followed by the extraction of RNA-seq reads that are uniquely aligned to these regions. Putative terminal exons are subsequently constructed based on the read alignment, with skipped terminal exons being defined by spliced reads and composite terminal exons being deduced by unspliced reads. Ultimately, a trained classifier is employed to determine the true terminal exon by computing features characterizing read alignment.

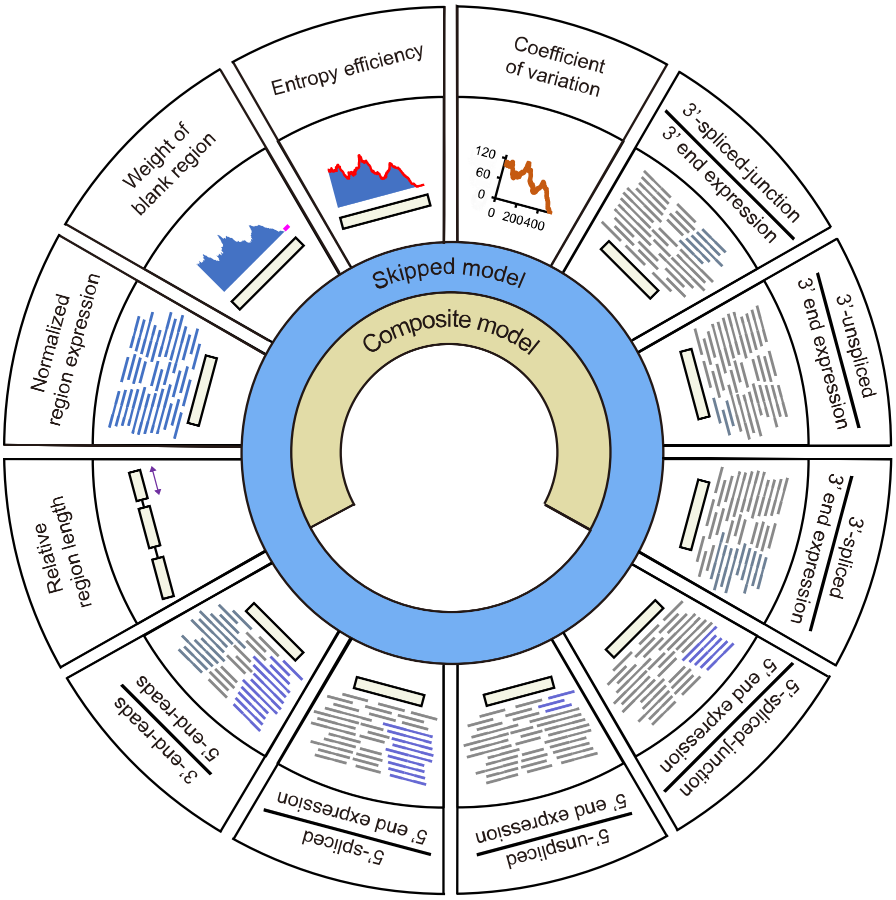

Supplementary Figure 3. Features utilized in the read module.

A schematic illustration of the features characterizing the read alignment, which are used to construct the random forest model to classify the intronic terminal exons form internal exons and background. To account for the differences between skipped and composite terminal exons, all twelve features are utilized in the model for skipped terminal exons (skipped model), while the model for composite terminal exons (composite model) employs eight different features as indicated.

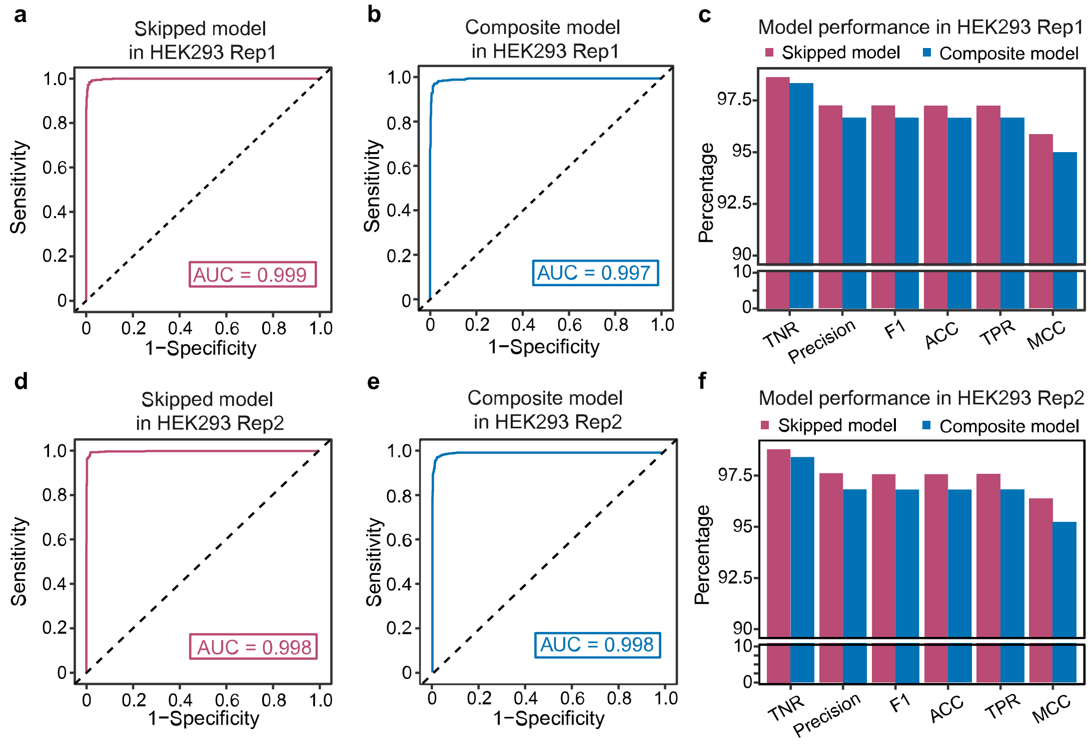

Supplementary Figure 4. Performance of the read module.

(a, b) The ROC curves show the performance on testing set of the skipped **(a)** and composited model **(b)** trained in HEK293 replicate1 dataset. The AUC is indicated in each plot.

(c) The bar plot depicts other metrics for evaluating the performance of the skipped and composite model trained in HEK293 replicate 1 dataset, including true negative rate (TNR), true positive rate (TPR), Accuracy (ACC), Precision, F1 score and Matthew's correlation coefficient (MCC).

(d, e) The ROC curves show the performance on testing set of the skipped **(d)** and composited model **(e)** trained in HEK293 replicate2 dataset. The AUC is indicated in each plot.

(f) The bar plot depicts other metrics for evaluating the performance of the skipped and composite model trained in HEK293 replicate2 dataset.

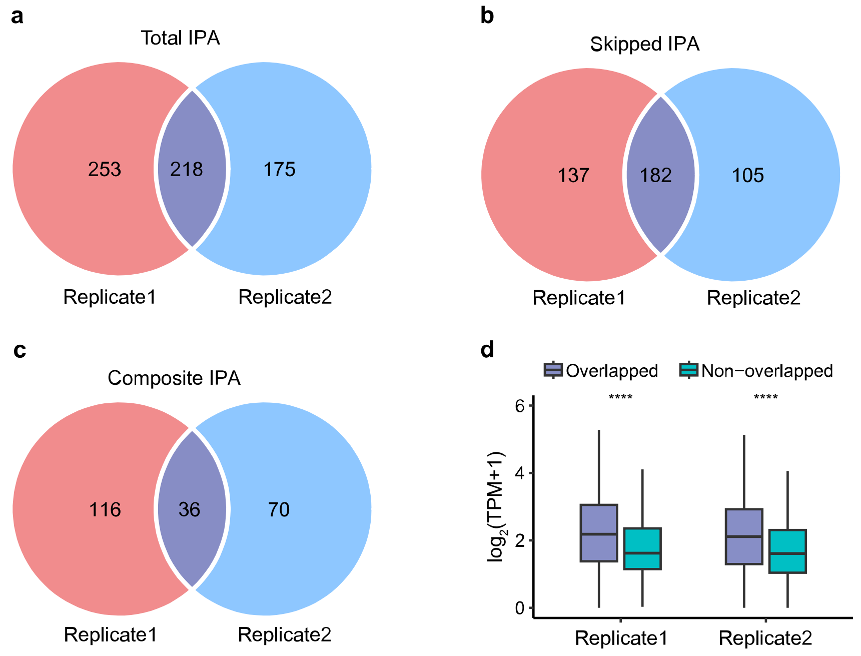

Supplementary Figure 5. Novel IPA sites identified in the two replicate HEK293 datasets using InPACT.

(a-c) The Venn diagram show the overlap of novel IPA sites identified in HEK293 replicate1 and replicate2 datasets (**a**). Two different types of IPA sites were compared separately, including skipped (**b**) and composite IPA sites (**c**).

(d) The comparison of the gene expression level between overlapped (n = 218) and non-overlapped (428) sites. The two-sided Wilcoxon rank sum test *P*-values are shown. The center lines denote the median values with the boxes are bounded by the 25th and 75th percentiles. The whiskers extend to the maximum and minimum values within 1.5 times the interquartile range (IQR) from each end of the box. **** *P*-value < 0.0001.

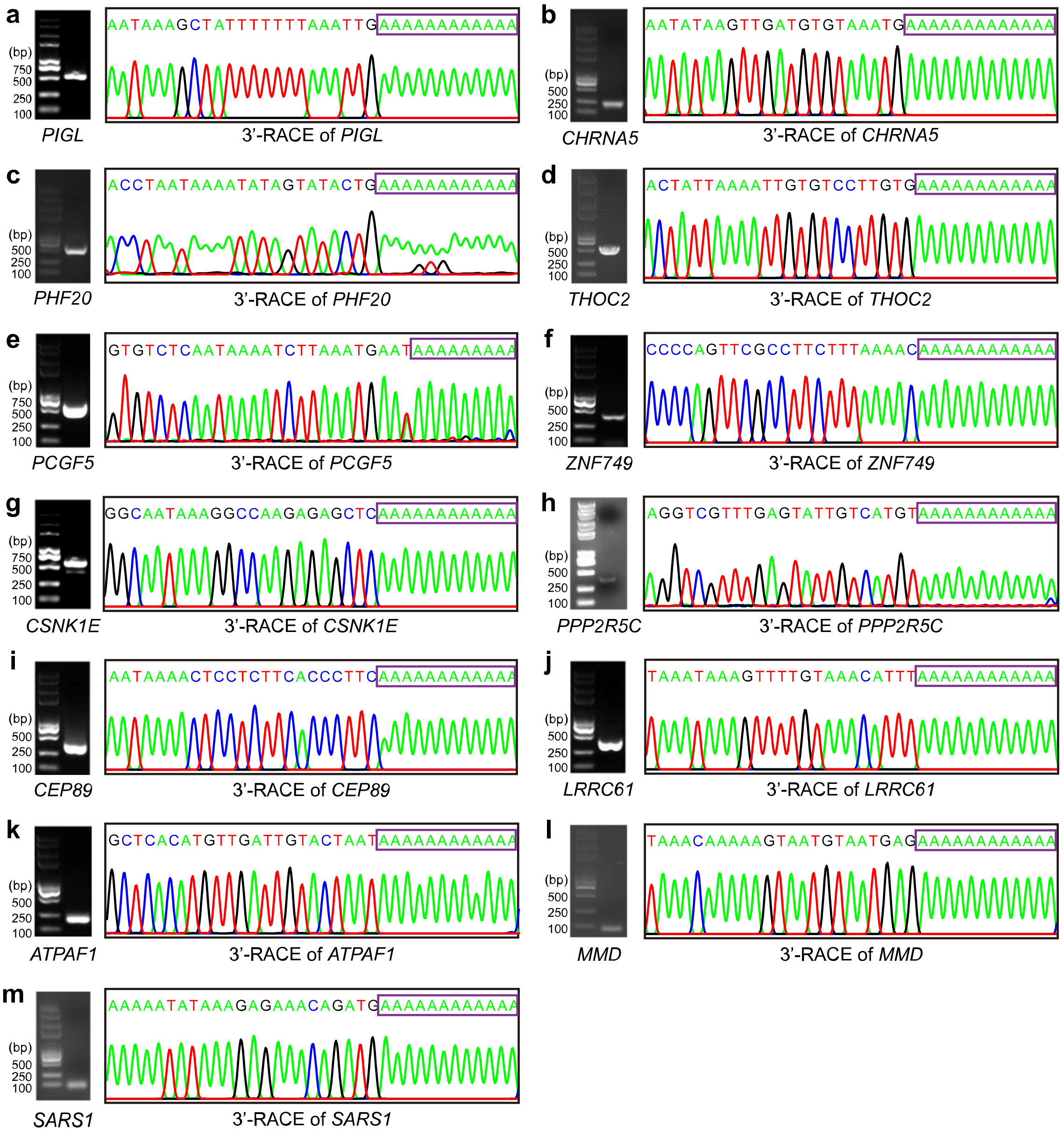

Supplementary Figure 6. Experimental validation of candidate IPA sites identified by InPACT in HEK293 cells

The gel of 3'-Rapid Amplification of cDNA Ends (3'-RACE) experiments and Sanger sequencing results of amplified transcripts by 3'-RACE experiments were depicted. The results of gene *PIGL* (**a**), *CHRNA5* (**b**), *PHF20* (**c**), *THOC2* (**d**), *PCGF5* (**e**), *ZNF749* (**f**), *CNSK1E* (**g**), *PPP2R5C* (**h**), *CEP89* (**i**), *LRRC61* (**j**), *ATPAF1* (**k**), *MMD* (**l**), and *SARS1* (**m**) were shown. All candidate IPA sites were confirmed in HEK293 cells. Each experiment was repeated n = 3 times.

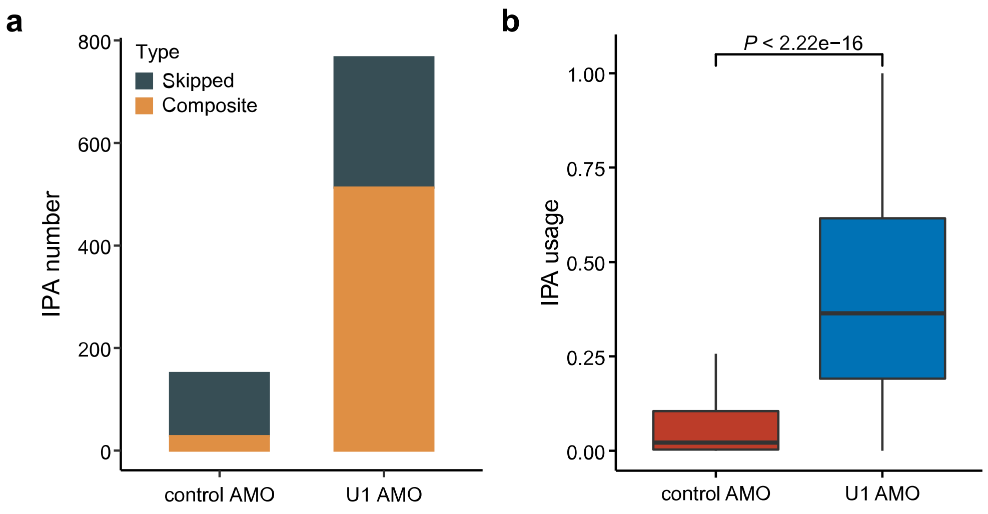

Supplementary Figure 7. IPA analysis of the RNA-seq datasets of HeLa cells treated with control and U1 AMO.

(a) The bar chart presents the number of IPA events predicted by InPACT in both the control and U1 antisense morpholino oligonucleotide (AMO) groups.

(b) The box plot depicts the distribution of IPA usage in the control and U1 AMO group (n = 870 IPA events in each group). The two-sided Wilcoxon rank sum test *P*-value is shown. The center lines denote the median values with the boxes are bounded by the 25th and 75th percentiles. The whiskers extend to the maximum and minimum values within 1.5 times the interquartile range (IQR) from each end of the box.

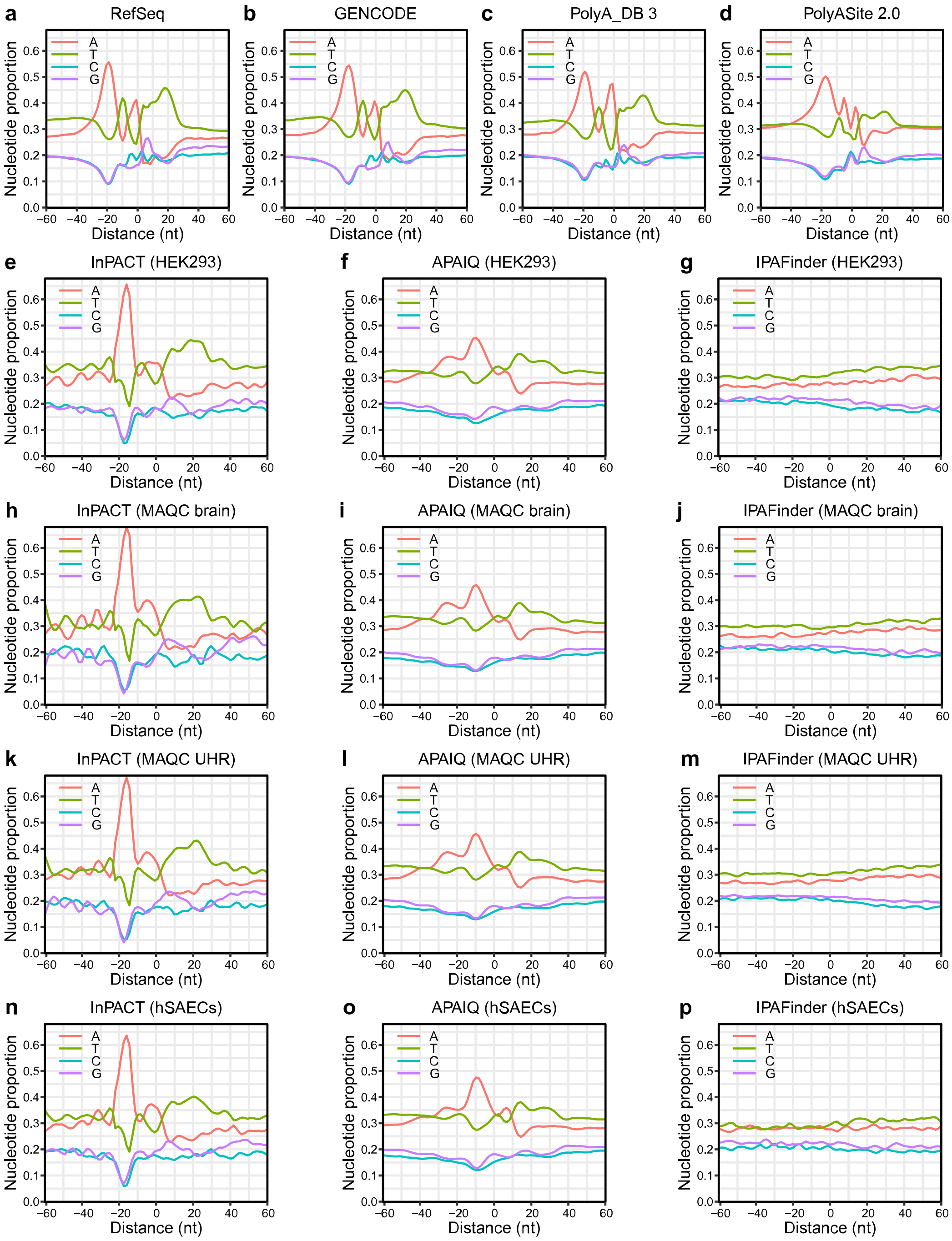

Supplementary Figure 8. Nucleotide compositions of the sequences surrounding IPA sites identified by InPACT, APAIQ and IPAFinder.

(a-d) In supplement to Figure 3A, the line plots show nucleotide compositions from upstream 50 nt to downstream 50 nt of the polyA sites from RefSeq (a), GENCODE (b), PolyA_DB 3 (c) and PolyASite 2.0 (d). The X-axis denotes the distance from upstream 50 nt to downstream 50 nt of those polyA sites. The Y-axis denotes the nucleotide fraction at each position.

(e-g) The comparison of IPA sites identified in HEK293 cells by InPACT, APAIQ and IPAFinder.

(h-j) The comparison of IPA sites identified in MAQC brain samples by InPACT, APAIQ and IPAFinder.

(k-m) The comparison of IPA sites identified in MAQC Universal Human Reference (UHR) samples by InPACT, APAIQ and IPAFinder.

(n-p) The comparison of IPA sites identified in human small airway epithelial cells (hSAECs) by InPACT, APAIQ and IPAFinder.

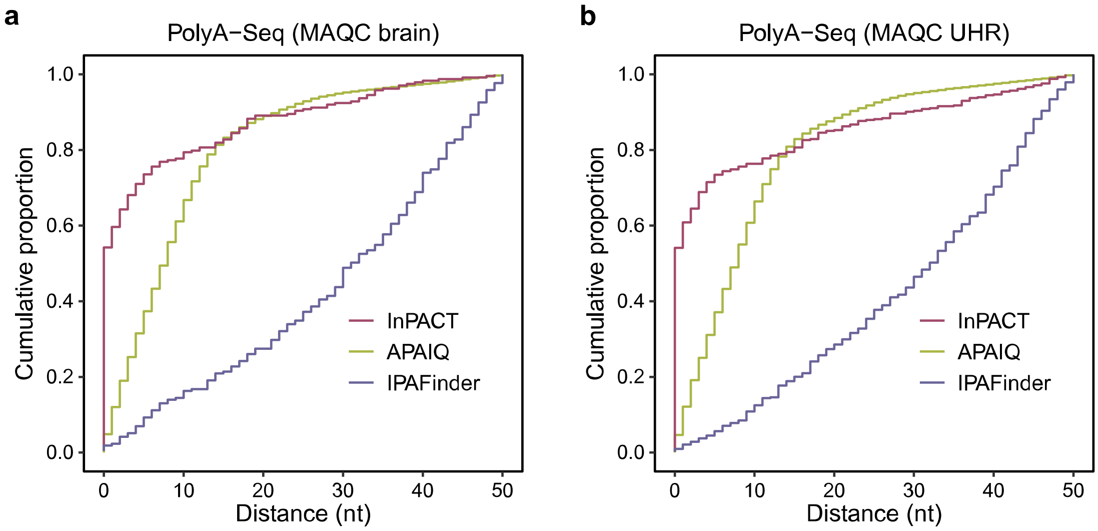

Supplementary Figure 9. InPACT demonstrates superior performance in Identifying IPA sites in the MAQC UHR and human brain datasets.

In supplement to Figure 3. The cumulative distribution curves of the distance between the true positive identified IPA sites and the ground truth obtained from PolyA-seq. If an identified IPA site is located within 50 nt from the ground truth, it was regarded as true positive. The datasets utilized for this analysis originate from MAQC brain (**a**) and UHR (**b**) samples.

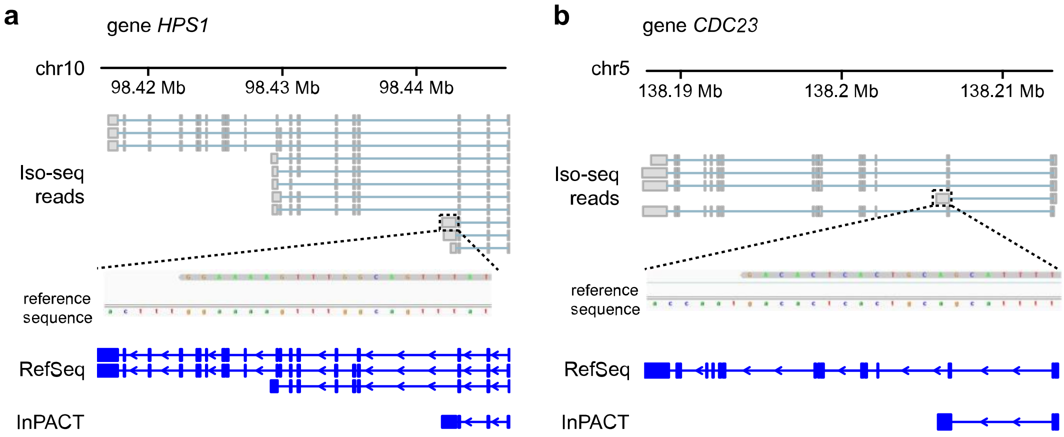

Supplementary Figure 10. The Iso-seq reads support the presence of IPA sites within gene *HPS1* and *CDC23* identified from RNA-seq data of human small airway epithelial cells.

(a, b) In supplement to Figure 3E, F. Genome browser view of *HPS1* (a) and *CDC23* (b) genes showing high quality transcripts from Iso-seq. Zoom-in shows the terminal sequences of Iso-seq reads, which are in accordance with the InPACT-predicted isoforms. The bottom part shows the annotated transcripts from RedSeq and assembled IPA isoforms from InPACT.

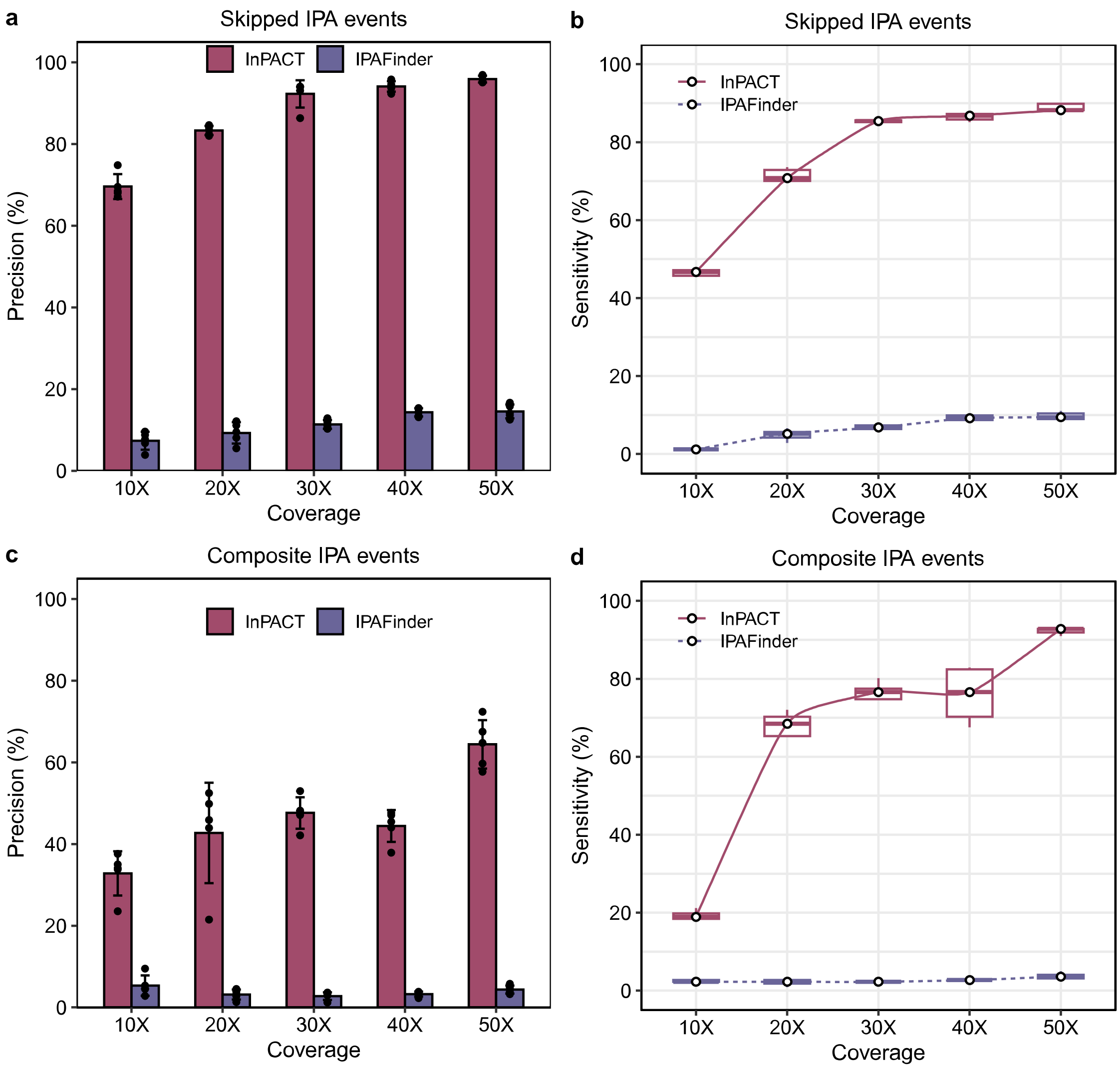

Supplementary Figure 11. Benchmarking of InPACT on identifying skipped and composite IPA using simulated RNA-seq datasets.

In supplement to Figure 3g and 3h. The precision and sensitivity of InPACT and IPAFinder are evaluated for identifying skipped **(a, b)** and composite **(c, d)** IPA sites using simulated RNA-seq data with varying sequencing coverage levels ranging from 10X to 50X. Replicates were utilized for each coverage level (n = 5 random simulations). The precisions are presented as mean values +/− SD. The sensitivities are presented as box plots. The center horizontal line in the box denotes the median with the bottom and top of the box show the 25th and 75th percentiles. The whiskers extend 1.5 times the interquartile range (IQR) from the top and bottom of the box.

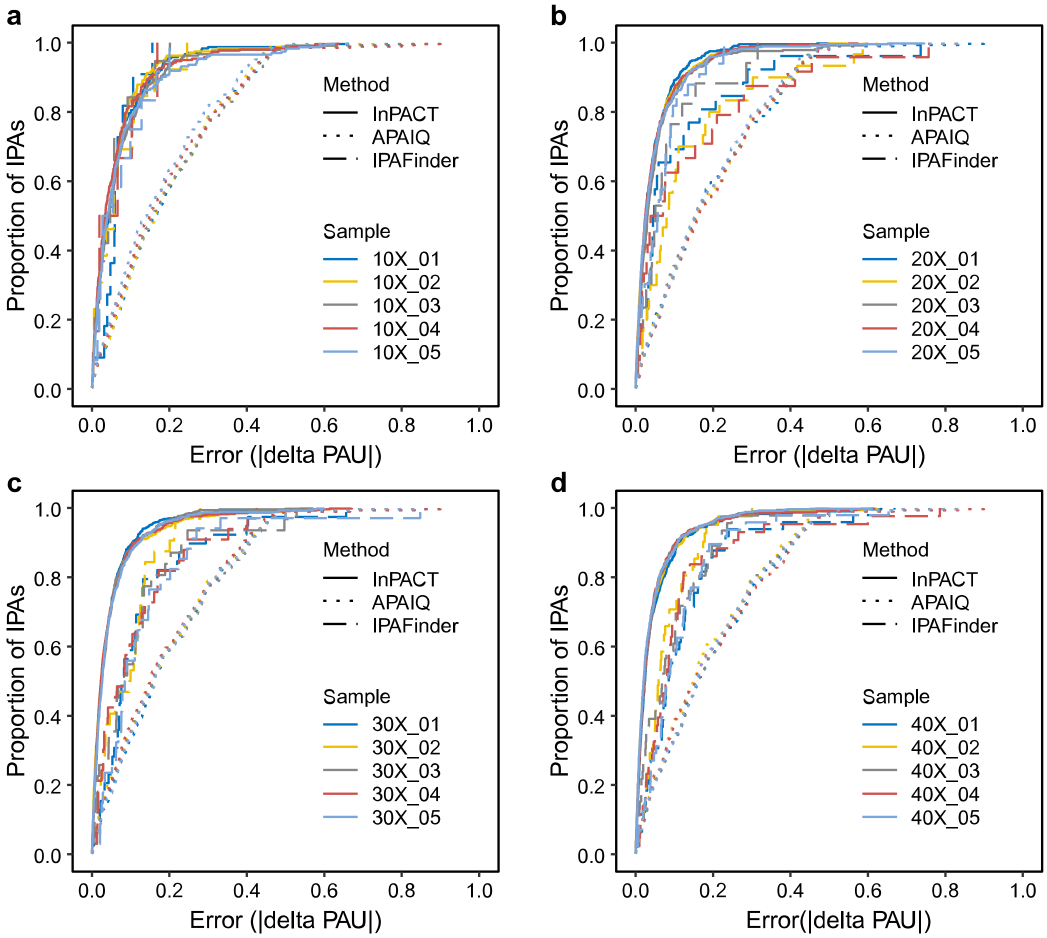

Supplementary Figure 12. Benchmarking of InPACT on IPA quantification.

In supplement to Figure 3i, the cumulative distribution curves show error of the relative usage of IPA sites determined by InPACT, APAIQ, and IPAFinder in those simulated RNA-seq data with varying sequencing coverage levels ranging from 10X to 40X.

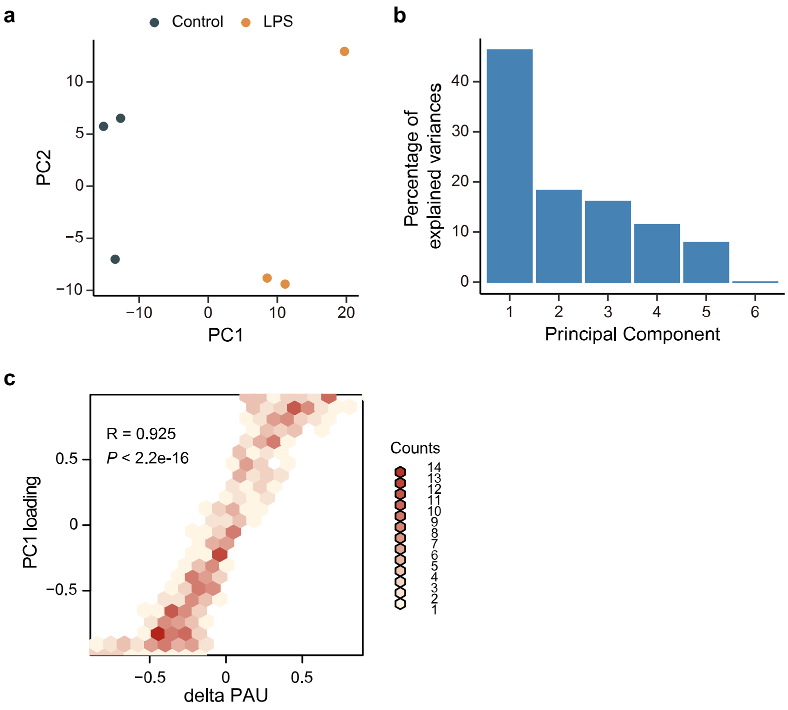

Supplementary Figure 13. Principal component analysis of the profile of IPA usage estimated on the RNA-seq data of untreated and LPS-activated human monocytes.

(**a**) The scatterplot shows the projections of the profile of IPA suage onto first (PC1) and second (PC2) principal component. The replicates of untreated and LPS-activated human monocytes are shown by colors.

(b) The bar plot depicts the proportion of variance explained by each estimated principal component.

(c) The hexbin scatterplot compares the change of IPA usage (n = 500) between untreated and LPS-activated monocytes and the corresponding estimated PC1 loadings. The Pearson correlation coefficient and its corresponding *P*-value are presented.

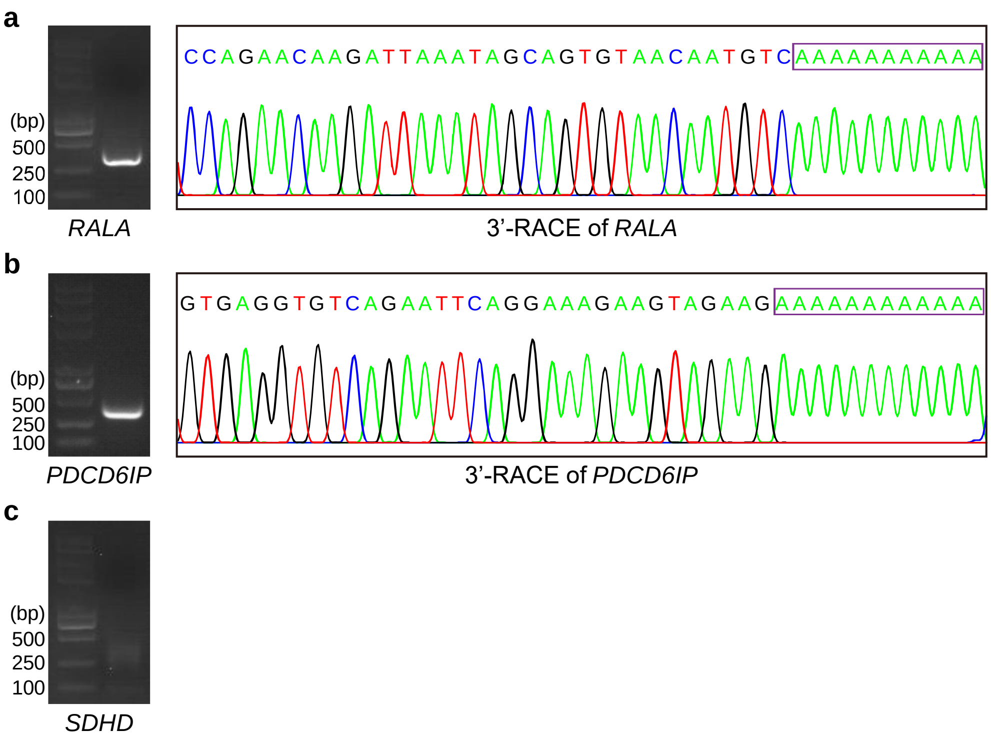

Supplementary Figure 14. Experimental validation of candidate IPA sites identified by InPACT in monocytes.

(a-b) The mRNA from monocytes was extracted, and then 3’-Rapid Amplification of cDNA Ends (3’-RACE) experiments were performed. The gel of 3’-RACE experiments (left) and Sanger sequencing results (right) for gene *RALA* (**a**) and *PDCD6IP* (**b**) were depicted. Each experiment was repeated n = 3 times.

(c) No clear band were detected in the gel of 3’-RACE experiment for gene *SDHD*. The experiment was repeated n = 3 times.
