# Supplementary material for: InPACT: a computational method for accurate characterization of intronic polyadenylation from RNA sequencing data
Source: Nat Commun. 2024 Mar 22;15:2583. doi: 10.1038/s41467-024-46875-8 (PMC10960005; doi:10.1038/s41467-024-46875-8)
Supplement: Supplementary file 2 — Description of Additional Supplementary Files [file 41467_2024_46875_MOESM2_ESM.pdf]

Description of Additional Supplementary Files

File Name: Supplementary Data 1

Description: Collection of sequencing datasets used in this study.

File Name: Supplementary Data 2

Description: Candidate IPA sites for experimental validation in HEK293 cells.

File Name: Supplementary Data 3

Description: Differential IPA events between untreated and LPS-activated human monocytes. DRIMSeq is used for differential analysis, and P-values are adjusted with the Benjamini-Hochberg method.

File Name: Supplementary Data 4

Description: Novel IPA sites identified in human FBM scRNA-seq data by InPACT.

File Name: Supplementary Data 5

Description: Cell type-specific IPA events identified for each cell type human FBM scRNA-seq data.

File Name: Supplementary Data 6

Description: The primer sequences for 3'-RACE experiments in HEK293 cells.

File Name: Supplementary Data 7

Description: The primer sequences for 3'-RACE experiments in LPS-activated monocytes.
