# Supplementary material for: InPACT: a computational method for accurate characterization of intronic polyadenylation from RNA sequencing data
Source: Nat Commun. 2024 Mar 22;15:2583. doi: 10.1038/s41467-024-46875-8 (PMC10960005; doi:10.1038/s41467-024-46875-8)
Supplement: Supplementary file 10 — Reporting Summary [file 41467_2024_46875_MOESM10_ESM.pdf]

nature portfolio

Corresponding author(s): Yang Yang

Last updated by author(s): Feb 26, 2024

Reporting Summary

Nature Portfolio wishes to improve the reproducibility of the work that we publish. This form provides structure for consistency and transparency in reporting. For further information on Nature Portfolio policies, see our Editorial Policies and the Editorial Policy Checklist.

Statistics

For all statistical analyses, confirm that the following items are present in the figure legend, table legend, main text, or Methods section.

n/a	Confirmed	
☐	☒	The exact sample size (*n*) for each experimental group/condition, given as a discrete number and unit of measurement
☐	☒	A statement on whether measurements were taken from distinct samples or whether the same sample was measured repeatedly
☐	☒	The statistical test(s) used AND whether they are one- or two-sided<br>*Only common tests should be described solely by name; describe more complex techniques in the Methods section.*
☐	☒	A description of all covariates tested
☐	☒	A description of any assumptions or corrections, such as tests of normality and adjustment for multiple comparisons
☐	☒	A full description of the statistical parameters including central tendency (e.g. means) or other basic estimates (e.g. regression coefficient) AND variation (e.g. standard deviation) or associated estimates of uncertainty (e.g. confidence intervals)
☐	☒	For null hypothesis testing, the test statistic (e.g. *F*, *t*, *r*) with confidence intervals, effect sizes, degrees of freedom and *P* value noted<br>*Give P values as exact values whenever suitable.*
☒	☐	For Bayesian analysis, information on the choice of priors and Markov chain Monte Carlo settings
☒	☐	For hierarchical and complex designs, identification of the appropriate level for tests and full reporting of outcomes
☐	☒	Estimates of effect sizes (e.g. Cohen's *d*, Pearson's *r*), indicating how they were calculated

Our web collection on statistics for biologists contains articles on many of the points above.

Software and code

Policy information about availability of computer code

Data collection	prefetch (v3.0.0) and wget (v1.19.5)
Data analysis	Python packages: h5py (v2.10.0), Keras (v2.0.6), tensorflow (v1.3.0), pandas (v1.1.5), numpy (v1.19.5), pybedtools (v0.9.0), HTSeq (v0.9.1), scikit-learn (v0.24.2), imbalanced-learn (v0.8.1), pyfasta (v0.5.2). Softwares: bedtools (v2.30.0), samtools (v1.14), subread (v2.0.3), salmon (v1.10.1), HISAT2 (v2.1.0), Ribowave (v1.0), STAR (v2.7.10a), STARlong (v2.7.4a), bigWigAverageOverBed (v2), IPAFinder (v1.0), APAIQ (v1.2), DeepPASS (v1.0), APARENT (v1.0), DeepPASTA (v1.0), MEME suite (v5.5.4) (https://meme-suite.org/meme/). R packages: polyester (v1.9.7), ggplot2 (v3.3.5), ClusterProfiler (v3.18.1), Gviz (v1.34.1), Rtsne (v0.15), DESeq2 (v1.30.0), tximport (v1.18.0), DRIMSeq (v1.18.0), stageR (v1.12.0), sangerseqR (v1.26.0). InPACT can be obtained from GitHub repository (https://github.com/YY-TMU/InPACT).

For manuscripts utilizing custom algorithms or software that are central to the research but not yet described in published literature, software must be made available to editors and reviewers. We strongly encourage code deposition in a community repository (e.g. GitHub). See the Nature Portfolio guidelines for submitting code & software for further information.

Data

Policy information about availability of data

All manuscripts must include a data availability statement. This statement should provide the following information, where applicable:

- Accession codes, unique identifiers, or web links for publicly available datasets
- A description of any restrictions on data availability
- For clinical datasets or third party data, please ensure that the statement adheres to our policy

The human reference genome GRCh38 was obtained from the UCSC genome browser (https:/genome.ucsc.edu). A detailed description of sequencing datasets used in this study is provided in Supplementary Data 1. Specially, the RNA-seq, ribo-seq, A-seq, and 3P-seq of HEK293 cells can be downloaded from GEO under accession number GSE56010 [https://www.ncbi.nlm.nih.gov/geo/query/acc.cgi?acc=GSE56010], GSE73136 [https://www.ncbi.nlm.nih.gov/geo/query/acc.cgi?acc=GSE73136], GSE37037 [https://www.ncbi.nlm.nih.gov/geo/query/acc.cgi?acc=GSE37037], GSE52527 [https://www.ncbi.nlm.nih.gov/geo/query/acc.cgi?acc=GSE52527], respectively. The RNA-seq dataset of HeLa cells treated with control and U1 AMO can be downloaded from GEO under accession number GSE193200 [https://www.ncbi.nlm.nih.gov/geo/query/acc.cgi?acc=GSE193200]. The RNA-seq and polyA-seq datasets of MAQC UHR and human brain can be downloaded from GEO under accession number GSE49712 [https://www.ncbi.nlm.nih.gov/geo/query/acc.cgi?acc=GSE49712] and GSE30198 [https://www.ncbi.nlm.nih.gov/geo/query/acc.cgi?acc=GSE30198], respectively. The RNA-seq and matched PacBio SMRT Iso-seq dataset of human small airway epithelial cells can be downloaded from GEO under accession number GSE167486 [https://www.ncbi.nlm.nih.gov/geo/query/acc.cgi?acc=GSE167486]. The RNA-seq dataset of untreated and LPS-activated human monocytes can be downloaded from GEO under accession number GSE118165 [https://www.ncbi.nlm.nih.gov/geo/query/acc.cgi?acc=GSE118165]. The scRNA-seq data of human fetal bone marrow can downloaded from EMBL-EBI ArrayExpress under accession number E-MTAB-9801 [https://www.ebi.ac.uk/biostudies/arrayexpress/studies/E-MTAB-9389].

Research involving human participants, their data, or biological material

Policy information about studies with human participants or human data. See also policy information about sex, gender (identity/presentation), and sexual orientation and race, ethnicity and racism.

Reporting on sex and gender	The sex or gender was not considered in the study design.
Reporting on race, ethnicity, or other socially relevant groupings	The race and ethnicity were not applicable to our analyses.
Population characteristics	Healthy donors were recruited
Recruitment	Healthy donors were recruited
Ethics oversight	This study complies with all relevant ethical regulations. Ethical approval of all human studies was obtained from the Ethics Committee of Blood Diseases Hospital, Chinese Academy of Medical Sciences. The relevant informed consent document was signed by the subject before sample collection and data acquisition.

Note that full information on the approval of the study protocol must also be provided in the manuscript.

Field-specific reporting

Please select the one below that is the best fit for your research. If you are not sure, read the appropriate sections before making your selection.

☒ Life sciences ☐ Behavioural & social sciences ☐ Ecological, evolutionary & environmental sciences

For a reference copy of the document with all sections, see nature.com/documents/nr-reporting-summary-flat.pdf

Life sciences study design

All studies must disclose on these points even when the disclosure is negative.

Sample size	The datasets analyzed in this study were obtained from publicly available datsets. Details regarding the sample size were provided in the Methods section of the paper. The study mainly utilized two HEK293 RNA-seq datasets, and three replicate RNA-seq datasets from untreated and LPS-activated human monocytes. The sample sizes for this study were chosen based on the availability of suitable datasets and the feasibility of conducting the analyses. Given the nature of the study and the availability of publicly available datasets, the selected sample sizes were sufficient to achieve the study objectives.
Data exclusions	No data was excluded.
Replication	Replication was incorporated: 1) N = 2 HEK293 RNA-seq datasets were used to evaluate the InPACT method, 2) N = 5 simulated RNA-seq data replicated for each coverage level were generated for benchmarking InPACT, and 3) N = 3 replicate RNA-seq data of untreated and LPS activated human monocytes were included to identify dynamic IPA events. 4) N = 3 experimental validation for each candidate IPA event have been conducted. And we confirmed all findings are reproducible.
Randomization	The InPACT model was constructed with the assurance of randomization through the implementation of random training and testing split.

Blinding	The blinding was not applicable to our analyses. The study was designed to provide a precise sample-wise characterization of IPA based on RNA-seq data. This focus on objective data analysis made blinding unnecessary for this aspect of the research.

Reporting for specific materials, systems and methods

We require information from authors about some types of materials, experimental systems and methods used in many studies. Here, indicate whether each material, system or method listed is relevant to your study. If you are not sure if a list item applies to your research, read the appropriate section before selecting a response.

Materials & experimental systems

n/a	Involved in the study
☒	☐ Antibodies
☐	☒ Eukaryotic cell lines
☒	☐ Palaeontology and archaeology
☒	☐ Animals and other organisms
☒	☐ Clinical data
☒	☐ Dual use research of concern
☒	☐ Plants

Methods

n/a	Involved in the study
☒	☐ ChIP-seq
☒	☐ Flow cytometry
☒	☐ MRI-based neuroimaging

Eukaryotic cell lines

Policy information about cell lines and Sex and Gender in Research

Cell line source(s)	HEK293T cells were obtained from the National Infrastructure of Cell Line Resource (1101HUM-PUMC000010, China).
Authentication	HEK293T cells were authenticated by examining their morphology and growth characteristics.
Mycoplasma contamination	HEK293T cells were tested for Mycoplasma contamination every 2 months during culture as routine test in cell laboratory using Mycoplasma Detection Kit (D101, Vazyme Biotech Co., Ltd) to ensure that the cells were Mycoplasma free.
Commonly misidentified lines (See ICLAC register)	None of the commonly misidentified cell lines were employed in this study.
